# Reliability of Frontal Eye Fields Activation and Very Low-Frequency Oscillations Observed during Vergence Eye Movements: an fNIRS Study

**DOI:** 10.1038/s41598-020-57597-4

**Published:** 2020-01-20

**Authors:** Chang Yaramothu, Xiaobo Li, Cristian Morales, Tara L. Alvarez

**Affiliations:** Department of Biomedical Engineering, New Jersey Institute of Technology, Newark, NJ USA

**Keywords:** Neural circuits, Visual system

## Abstract

Functional near-infrared spectroscopy (fNIRS), an imaging tool that utilizes infrared light to measure changes within the concentration of oxygenated (HbO) and deoxygenated (HbR) hemoglobin, holds promise to study functional activity from motor, visual, and memory cortical regions using stimulus-induced tasks. This study investigated the reliability for fNIRS to examine cortical activations within the frontal eye fields (FEF) while initiating vergence eye movements, the inward and outward rotation of the eyes. FNIRS data were collected from twenty participants with normal binocular vision while performing vergence eye movements compared to sustained gaze fixation within a block design during two different sessions. Reliability of the experimental protocol was assessed using the intraclass correlation coefficient (ICC). The ICC values ranged from 0.6 to 0.7 for measuring the HbO activation within the vicinity of the FEF. A frequency power spectrum analysis revealed two predominant frequencies within the functional activation signals from the FEF. One high-intensity signal was present at 0.029 Hz, centering around the block design frequency. The peak-intensity signal was observed between 0.012 and 0.018 Hz where this very low-frequency oscillation (VLFO) was hypothesized to be generated by the macrovasculature present near the FEF and should be avoided as a block design frequency in future fNIRS studies to avoid false positive results.

## Introduction

Functional near-infrared spectroscopy (fNIRS) is a non-invasive functional imaging tool. FNIRS capitalizes on the different absorption characteristics of oxygenated (HbO) and deoxygenated (HbR) hemoglobin when exposed to 600–900 nm infrared light to detect individual differences of HbO and HbR concentrations^1,2^. The scattered and re-emitted infrared light is measured by a detector placed between 10–40 mm from a source^3–6^. This source-detector distance induces a light penetration depth of 2–8 mm into the cortex^7^. The proposed brain region is located within the vicinity of the centroid between the source and detector optode, roughly 15 mm below the scalp^8^. Scattering of light creates an elliptical shaped pathway between the optodes, making the observed signal an aggregate of that pathway^9^. This collective signal contains the hemodynamic concentrations from the neuro-vasculature, grey matter and white matter.

For the scattering and recapture of infrared light, optimal optode placement requires that the optodes have direct contact with a participant’s scalp. Anything less than an orthogonal contact by the optode with the scalp will result in extra-cranial scattering of light, creating noise artifacts within the collected data^5^. A high signal-to-noise ratio (SNR) is attained by housing the optodes in a holder called a head cap. The head cap, its design, and material composition are very important for the optimal collection of data. An optimization of the design results in more optical pathways that can observe the hemodynamic concentrations within a specific region. To further improve signal quality, the material composition should allow for an easy attachment of the optodes to the head cap, simultaneously allowing the head cap with the optodes to contour to the shape of each participant’s scalp that will each have different anatomy.

Functional activation is observed in one or more regions of the brain while performing a sensory or cognitive task. The task for this study is vergence eye movements. Vergence eye movements are the inward and outward rotation of the eyes needed to perceive objects of interest as a single percept. Several studies indicate that the frontal eye fields (FEF), posterior parietal cortex (PPC), cerebellar vermis (CV) and brainstem are some of the neural substrates utilized to mediate a vergence response assessed via experimental studies using single cell recordings from macaque primates or functional MRI from humans^10–22^. Previous investigations are scant studying eye movements and its respective cortical activation with infrared spectroscopy where a thorough literature review did not reveal any prior peer-reviewed publications indexed in Scopus or PubMed. The FEF will be the only region of interest (ROI) for this study because the fNIRS instrumentation restricts the number of ROIs from which the data can be collected. Additionally, since the FEF is on the surface of the cortex, it is well poised to be an ROI that can emit a reliable signal to be studied using fNIRS.

Prior studies have calculated the fNIRS intraclass correlation coefficient (ICC) on various tasks such as, handgrip^23^, checkerboard visual stimulation^24^, finger tapping^25^, sustained attention task^26^ and resting-state functional connectivity^27,28^. A literature search did not reveal any studies that report an ICC for an oculomotor task using fNIRS studying the FEF. The clinical significance criteria established by prior studies states that the following ICC values: ICC < 0.4, 0.4 < ICC < 0.59, 0.60 < ICC < 0.74, and 0.75 < ICC < 1.00, are considered poor, fair, good, and excellent, respectively^27,29–35^. Prior imaging studies have also established a floor threshold ICC of 0.4, where a reliable ICC for most reasonable functional magnetic resonance imaging (fMRI) studies have a value between 0.33 and 0.66^29,36^. This study will utilize those prior established standards for assessment of the reliability of fNIRS. The goal of this investigation is to calculate the reliability of a stimulus-induced vergence eye movement task during an fNIRS experiment to determine its feasibility as an outcome measurement for future basic science studies and future randomized clinical trials.

## Results

All participants had normal binocular vision, see Supplementary Table 1 for all binocular vision measurements. Eye movements were examined to ensure compliance. All the participants performed vergence eye movements to symmetrical step stimuli during the task phases and sustained fixation during the rest phases.

Classification of reliability of the fNIRS signal originating from the FEF was assessed through ICC values. The ICC values were calculated by measuring the four hemodynamic channels directly over the FEF: specifically channels 2, 5, 10, and 13. The ICC values of oxygenated (HbO), deoxygenated (HbR), and total (HbT) hemoglobin modalities are listed in Table 1. The ICC values ranged from 0.60 to 0.70, 0.32 to 0.51, and 0.68 to 0.71 for HbO, HbR, and HbT, respectively. Based on the observed values, the HbO and HbT signals have good reliability, while the HbR signals have poor to fair reliability on average. Since HbT is a combination of the HbO and HbR, only statistical comparisons between HbO and HbR were calculated. A paired t-test comparing the HbO and HbR ICC values was conducted for each channel. There was a significant difference between the HbO and HbR ICC for all of the following four channels: Ch2 [t(15) = 2.687; *p* = 0.017], Ch5 [t(12) = 2.245; *p* = 0.044], Ch10 [(t (12) = 2.447; *p* < 0.001), and Ch13 [t(14) = 3.676; *p* = 0.002].

**Table 1:** Group-Level ICC Values.

	Left FEF		Right FEF	
	Channel 2	Channel 5	Channel 10	Channel 13
HbO	0.60	0.62	0.70	0.61
HbR	0.51	0.49	0.32	0.42
HbT	0.71	0.68	0.69	0.67

**Figure 1:**
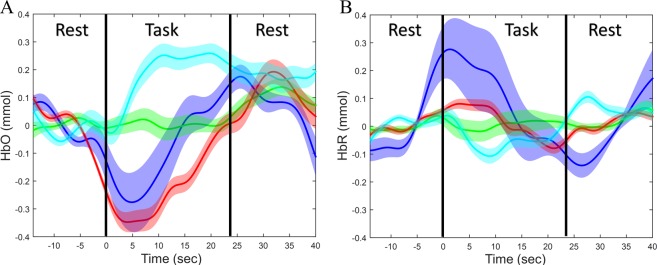
Group-level hemodynamic response functions (HRF) for the oxygenated (HbO, **A**) and deoxygenated (HbR, **B**) response signal. Group-level oxygenated hemodynamic response function (HRF) from 20 participants of the average of the 10 task / rest blocks of the acquired signals. Each of the colors in the HRF correspond to a different channel based on the source-detector design. Channel 2 from Source A to Detector 2 (blue line), Channel 5 from Source B to Detector 1 (green line), Channel 10 from Source C to Detector 6 (red line), and Channel 13 Source D to Detector 5 (cyan line). The corresponding shaded colors is one standard deviation from the mean signal.

The high ICC values of HbO and HbT between sessions 1 and 2 quantify the reliability of the fNIRS signal using the custom high strength multipurpose neoprene rubber head cap and vergence eye movement experimental task protocol. Figure 1 is the group-level hemodynamic response functions (HRF) for the oxygenated (HbO, Fig. 1A) and deoxygenated (HbR, Fig. 1B) response signal. Figure 1 is the composite average of the 10 seconds ‘rest block’ prior to the task, the 10 task blocks averaged with the 10 rest blocks after the task for each of the four channels over both the FEFs. The blue (channel 2) and green (channel 5) traces are from the averages for the left FEF. The red (channel 10) and cyan (channel 13) traces are from the right FEF. The shaded regions represent one standard deviation from the mean signal. The rest and task phases of the HRF are noted and separated by solid black lines. Figure 1A shows an initial decrease in HbO, followed by an increase which is sustained for a few seconds after the end of the task phase. Channel 2 has the fastest hemodynamic response while Channel 5 is more delayed compared to the composite of the four channels. Channels 10 and 13 exhibit a stronger decrease in functional activity compared to channels 2 and 5. The increase and decrease in the HbO is consistent with the block design of the experimental task and rest paradigm. Conversely, the the HbR (Fig. 1B) responses exhibit an inverse effect compared to the HbO responses. A decrease in the magnitude of the HbR signal compared to the HbO signal is observed as a general trend.

**Figure 2:**
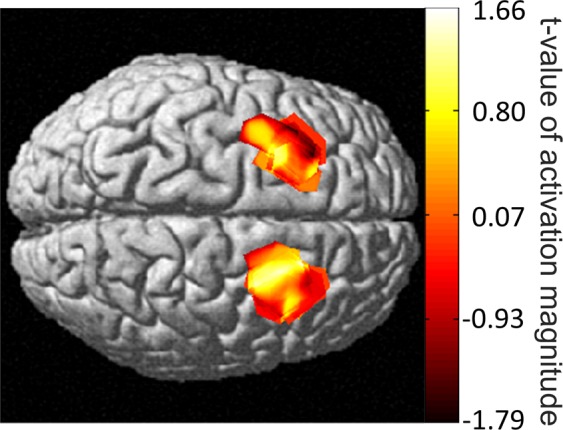
Group level HbO cortical activation map. The heat map shows the t-value of significant task-responsive BOLD activations.

Significant task-responsive group-level cortical activation analyzed through NIRS-SPM is shown in Fig. 2. The pixels which have significant activation are shown in Fig. 2 which is the signal t-value overlay on a model brain. The spatial resolution (area of significant cortical activation) slightly differed between participants. The time series and cortical activation maps showed feasibility to measure cortical activation from the FEF during vergence eye movements with fNIRS instrumentation.

**Figure 3:**
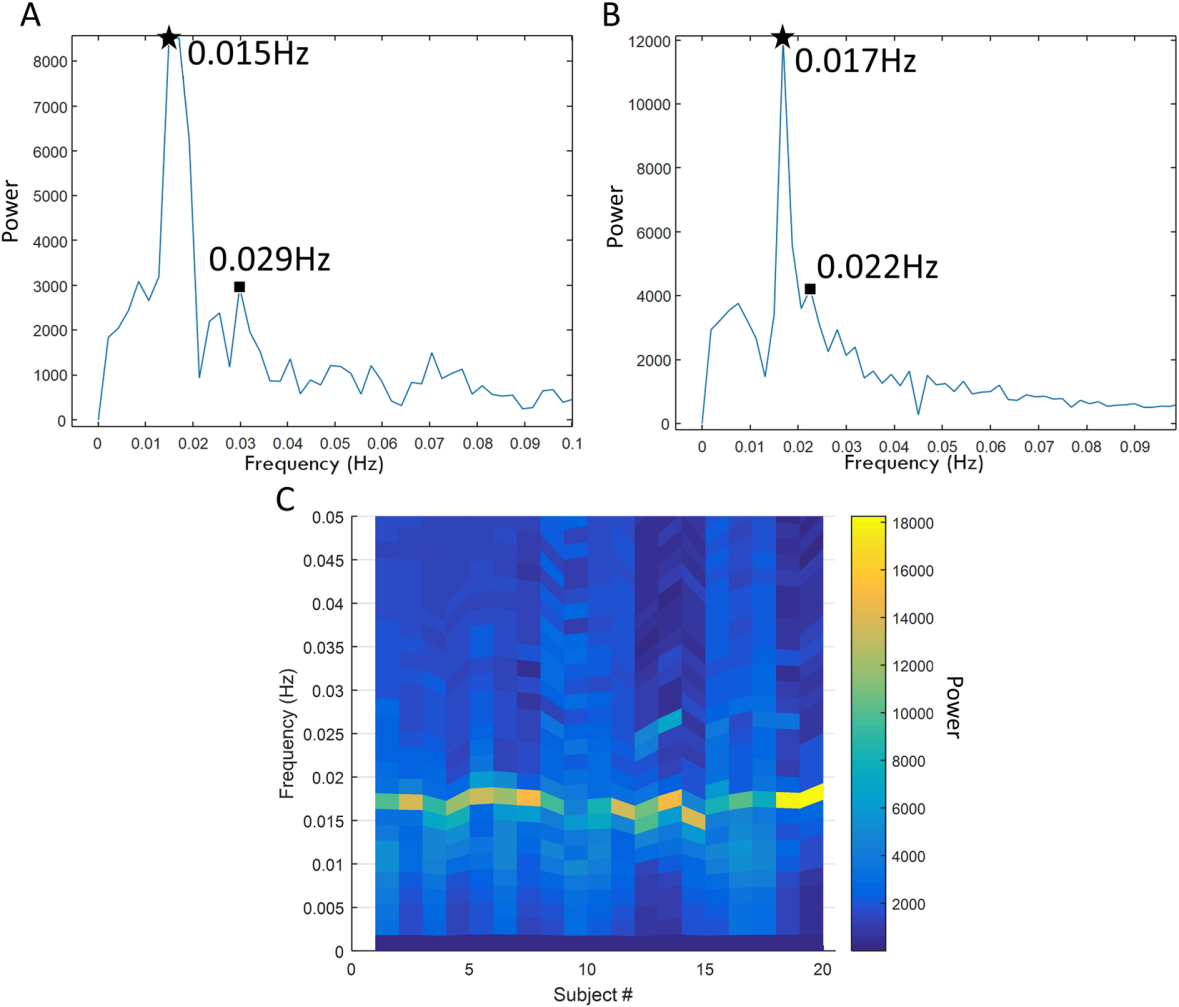
Frequency spectrum of HbO of Channel 2 for two different participants (plots **A**, **B**) showing the two peak-intensity signals, one denoted with a * and the other with a square. The experimental task has a frequency of 0.025 (**C**) shows the peak power and its corresponding frequency of all 20 participants.

The frequency analysis of the normalized HbO time series channels directly over the FEF ROI (Channels 2, 5, 10, and 13) yielded two frequencies with high intensities within the 0.01 and 0.1 Hz range. The frequency of the experimental design is 0.025 Hz, due to the fixation (rest) and eye movement (task) phases being 16 and 24 seconds long, respectively. The frequency of the HbO movement through the FEF, should be located approximately at this frequency. The data, on the contrary, shows a different prevalent frequency for the channels directly over the FEF. Figure 3A,B illustrate the frequency spectrum of channel 2 for two different participants which are representative for all participants. Figure 3C shows the frequency spectrum of all the subjects, where the power intensity is denoted by the color bar. Figure 3 shows that the two frequencies were of higher intensity compared to the bandwidth of frequencies observed, one with a high-intensity at approximately 0.016 Hz (denoted as a star in Fig. 3A,B) and the peak-intensity at approximately 0.025 Hz (denoted as a square in Fig. 3A,B). This trend is present in all four channels over the FEF, for all 20 participants. Supplementary Table 2 reports the frequency with the highest power for each subject. Table 2 reports the average of the frequencies with the two largest intensities for all the participants; one peak-intensity signal centered around 0.016 Hz and another high-intensity signal located approximately around 0.029 Hz. The range of these peak intensity signals for the 20 participants within the 0.01 and 0.1 Hz range are also listed in the Table 2. The peak intensity signal of the very low-frequency oscillator ranges from 0.012 to 0.018 Hz and was observed in all the 20 participants. The remaining three channels have a similar trend were one high-intensity signal was observed around 0.025 Hz and the peak-intensity signal was observed about 0.016 Hz.

**Table 2:** Group-level HbO Frequency Analysis average plus one standard deviation.

	Left FEF		Right FEF	
	Channel 2	Channel 5	Channel 10	Channel 13
Average of 1^st^ peak-intensity Frequency Component	0.016 ± 0.001 Hz	0.016 ± 0.001 Hz	0.016 ± 0.001 Hz	0.016 ± 0.001 Hz
Average of 2^nd^ peak-intensity Frequency Component	0.028 ± 0.005 Hz	0.029 ± 0.005 Hz	0.033 ± 0.004 Hz	0.035 ± 0.005 Hz
Highest Frequency Range	0.012–0.017 Hz	0.016–0.017 Hz	0.015–0.017 Hz	0.012–0.018 Hz

## Discussion

The high strength multi-purpose neoprene rubber was effective in all 20 participants. The elastance of the rubber allowed for the quick placement and removal of optodes from the cap. This elastance, additionally, did not compromise the position of the optodes in the cap. The cap’s flexibility aided in its ability to contour around the scalp to maintain each optode’s orthogonal contact to the scalp. The shape of the cap maintained the source-detector configuration and distances for the four channels used to measure functional activity over the bilateral FEFs.

The high ICC values demonstrate the efficacy of the high strength multipurpose neoprene rubber, as well as the reproducibility of the signals. There was, however, a significant difference between the HbO and HbR ICC values. The HbO signals had greater ICC values compared to the HbR signals. This difference can be explained with the following two main reasons. The CW6 (fNIRS Instrumentation) manufacturer, stated that the HbR will have a lower signal-to-noise ratio (SNR) compared to the HbO. Additionally, the visible cardiac wave in the raw data signal acquisition resulted in a noisier HbR signal compared to the HbO signal. This trend between the HbO and HbR signals has been reported in prior literature, where one study shows the HbO signal could be up to five times more pronounced than the HbR signal^37^. For this reason, it is common for most interpretations to be made from the HbO signals for fNIRS studies.

The HbT signal which is a summation of HbO and HbR had good ICC values. Each of the three modalities, HbR, HbO, and HbT represent different aspects of functional activity from the ROI. The HbO is correlated to the oxygen flowing into the tissue. HbR indicates the quantity of oxygen absorbed by the tissue, while, the HbT indicates the total blood flowing through the tissue^2^. Although each of the modalities convey different information, they all are related to the oxygen being consumed which is associated with the oxygen flowing into a specific tissue region. Hence, any of these three modalities are a metric of the metabolic activity to a ROI.

The hemodynamic response functions (HRF) generated from the time series of the HbO, HbR, and HbT correspond to the experimental protocol of a square function. However, the prior literature confirms that the HRF is participant and ROI dependent and variation is also observed to different experimental protocol tasks^38^. When a task has been initiated, the body responds via a demand for oxygenated blood to the functional region within the brain. The oxygenated blood around the ROI is the first to be used by the tissue, followed by the new oxygenated blood to the ROI due to the demand created by the task^39^. This study further supports that behavior as shown in Fig. 1. The channels directly over the bilateral FEF (channels 2, 5, 10, and 13) have an initial decrease in the HbO at the start of task, followed by an increase, and finally a decrease shortly after the end of the task. This trend exemplifies the initial uptake of the oxygen by the tissue, followed by the increased oxygenated blood flow to the FEF, and finally the blood flow returning to baseline during the rest task.

The frequency analysis of the HbO time series from the four primary long channels studying FEF revealed two high-intensity signals over two distinct frequencies. One high-intensity signal corresponded with the experimental task around 0.025 Hz and the peak intensity signal was observed between 0.012 to 0.018 Hz for the 20 participants studied. The channels directly over the FEF had two predominant frequencies, while the surrounding channels only had one (not shown in the result section in interest of brevity). A singular dominant frequency was hypothesized for all channels, centering around the task frequency; however, this study observed two high-intensity signals. The channels surrounding the FEF had a predominant frequency, approximately around the task frequency in addition to a separate peak intensity signal. The predominant peak frequency for the channels directly over the FEF was between 0.012 and 0.018 Hz. It is hypothesized that this very low-frequency oscillation (VLFO) detected in the fNIRS channels originated in the macrovasculature directly over the FEF ROI. Illustrations of the cranial vasculature by Netter show the precuneal artery traversing over the approximate location of the FEF^40^.

This study reports at frequency range of 0.012 to 0.018 Hz and proposes it may be due to macrovasculature. Previous studies proposes that skin blood flow oscillations corresponding to the endothelium-related metabolism generated a signal within the frequency range of 0.008–0.02 Hz^41,42^. Another study, attributes a frequency range between 0.02–0.046 Hz to skin blood flow oscillations which are those of neurogenic sympathetic nature^43^. These studies, in large, identify the vasculature under the skin as the mechanisms generating the VLFOs within this frequency range. Coupled with Netter’s illustrations, one interpretation of the VLFOs observed within this study of 0.012 to 0.018 Hz may be due to the macrovasculature found beneath the skin, and over the FEF cortical area. This VLFO is important to future fNIRS experiments studying the FEF because experimental paradigms which have a frequency between 0.012 and 0.018 could lead to a false positive signal generation since it would be unclear whether the signal is from the experimental task or the macrovasculature. One key result of this study is that our results suggest that researchers avoid designing an fNIRS task with a frequency between 0.012 and 0.018 Hz.

While causality between the VLFO signal and the microvasculature cannot be established within this current study, individual MRI, optical tomography, or angiography with contrast scans would be beneficial in identifying the precise vasculature under the RIOs to validate the presented theory. The data and concepts presented in this study show the viability of the fNIRS instrumentation and its reliability in measuring cortical activation in the FEF ROI while initiating vergence eye movements. The methods presented, additionally, show the viability of the integration between the eye tracking and fNIRS instrumentation. The fNIRS head cap designed for this study successfully secured the optodes while allowing the optodes to make orthogonal contact with the scalp. The success of the fNIRS head cap and experimental stimulus induced task design is quantified in the ICC values which would be categorized as good from established criteria. Finally, this study reports a peak intensity signal at a very low-frequency oscillator (VLFO) which was generating artifacts in the channels directly over the FEF. This VLFO is hypothesized to be generated by the macrovasculature overlaying the FEF.

## Materials and Methods

### Participants and inclusion criteria

A total of 20 participants (12 males) with a range between 20 and 34 years of age (mean 24.4 ± 3.5 years) participated in the study. All participants signed informed consent documentation prior to the experiments, which were approved by the New Jersey Institute of Technology’s (NJIT) Institutional Review Board (IRB) in accordance with the Declaration of Helsinki. All participants, had normal ocular health using methods described in previous vergence oculomotor studies which includes a battery of oculomotor tests^44–49^.

### Experimental setup

A haploscope system was used to stimulate vergence disparity, similar to the one shown in previous studies which keeps accommodative vergence constant^45,50^. Symmetrical disparity vergence step responses were presented to each eye via two separate monitors. Two partially reflective (50%) mirrors were used to present the eccentric square stimuli onto a participant’s field of view. The two mirrors were placed at a 90° to each other to ensure only stimuli to each eye were viewable to that eye. The participants and visual stimuli were carefully aligned to ensure all visual stimuli were presented on the participant’s midsagittal plane. The experimental space surrounding the participants was covered by commercial blackout curtains (Blackout Curtains, St. Louis Park, MN, USA) to minimize the amount of light emitted into the experimentation space and thus reduce proximal vergence cues. Participants verbally confirmed, prior to each experimental session, that no external light source was perceivable.

#### Eye tracking instrumentation

An ISCAN video-based eye tracking camera system (model ETL 400, ISCAN Inc., Burlington, MA, USA) was used to record eye movements. The manufacturer specifies that this infrared (λ = 950 nm) video-based system has an accuracy of 0.3° over a ± 20° horizontal range. Two cameras, one in front of the left-eye and the other in front of the right-eye placed at the manufacturer’s recommended distance of 15 inches, were used to capture oculus rotation. A board beam infrared source was used to illuminate the participant’s eyes. The maximum infrared light power level was 1.2 mW/cm^2^, which is well below the ANSI Z136 specification safety limit of 10 mW/cm^2^. The centroid of the pupil was used to quantify individual eye movements at the rate of 240 frames per second (fps). The cameras have a clear line of sight to the participant’s eyes and were not blocked by any materials including the partially reflective mirrors. Stimuli presentation and data acquisition were controlled by the VisualEyes2020 (VE2020) program, a custom LabVIEW™ 2013 SP 1 Virtual Instrument (National Instrument, Austin, TX, USA)^51^. Eye position data were digitized to ensure each participant performed the task.

#### fNIRS instrumentation

Hemodynamic changes were recorded with TechEn’s NIRSOptix Continuous Waveform 6 (CW6) (TechEn Inc., Milford, MA, USA). This system utilizes two infrared lasers, one at 690 nm and another at 830 nm for measuring deoxygenated and oxygenated hemoglobin concentration, respectively. The source and detector optodes are affixed to the scalp via a custom head cap described in detail below. The hemodynamic changes were quantified at 50 Hz and saved in an *. nirs format for offline analysis. A dark cloth was placed over the optodes to reduce any artifacts of infrared light from the eye-tracking instrumentation being collected by the fNIRS detectors.

#### Head cap

The material composition of the head cap had two main functions: (1) be flexible enough to easily insert and remove optodes from the head cap, while also having the durability to keep optodes attached to head cap for a long duration of time and (2) have the flexibility to contour around the different anatomies of each participant’s human head. Due to these requirements, soft plastics were more advantageous compared to hard plastics. A high strength multipurpose neoprene rubber (McMaster-Carr, Robbinsville, NJ, USA, part number: 8568K1) with 70A durometer sufficed both criteria above and was used for the head cap.

**Figure 4:**
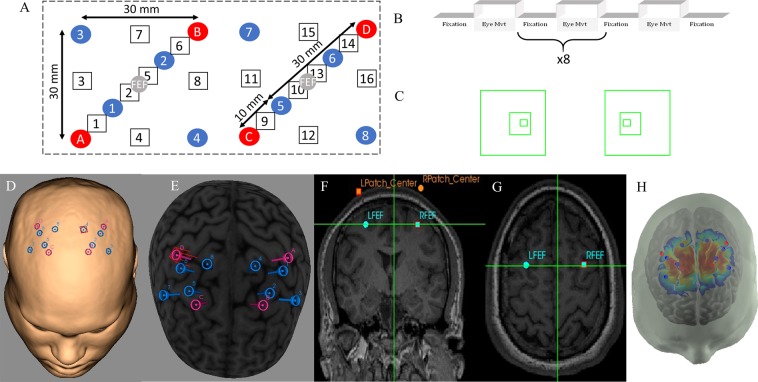
(**A**) Source-detector (SD) configuration, the source (red) and detector (blue) optodes are denoted as circle, functional channels are denoted as squares, and the FEF location is denoted by the gray FEF circles. All channels have 30 mm SD distance except for channels 1, 6, 9, and 14 which have an SD distance of 10 mm. (**B**) The experimental block design was composed of sustained fixation (denoted as Fixation) and vergence eye movements (denoted as Eye Mvt) which modulated functional activity within the vergence neural substrates. (**C**) Eccentric square stimuli; when converged upon, a person will perceive depth, where the squares will appear one in front of the other (larger to smaller). The source (pink) and detector (blue) optode locations on a skin (**D**) and cortical (**E**) overlay with the utilized FEF location in a coronal (**F**) and axial (**G**) view with corresponding optode patch location is show in the right side of the figure. The sensitivity map (**H**) was generated using AtlasViewer.

The source-detector (SD) design for the cap is important to maximize the number of channels that collect data, while minimizing the number of optodes used. The minimization of the number of optodes is required because of the physical restriction on how many optodes are available within the instrument and how many could be placed on the scalp within a given region. Figure 4A shows the source-detector design used for the study. Sources were labeled with letters (e.g. Source A), while the detectors were labeled with numbers (e.g. Detector 1). This compact design utilizes four sources for the entire experiment. This design additionally provides a total of six channels in each hemisphere that can study the functional activity of the FEF (SD distance of 30 mm) that allows the light to penetrate the cortex and can be called ‘long’ channels. In addition, two physiological channels (SD distance of 10 mm) are used to measure the superficial layers of predominantly the scalp also called ‘short’ channels. The data from the four physiological channels did not substantially improve the signal to noise ratio. In the interest of brevity, only the longer distance channels of 30 mm are presented within the results. The four channels directly over the FEF are channels 2 (Source A to Detector 2), 5 (Source B to Detector 1), 10 (Source C to Detector 6) and 13 (Source D to Detector 5) as shown in Fig. 4A.

#### Synchronization of fNIRS and eye movement tracking instruments

Our custom VE2020 software package is the central synchronization software for the entire experimental protocol. The scripts in VE2020 present the visual stimuli to the participant in the haploscope and synchronize the quantification of eye movement responses with the visual stimuli. VE2020 software is custom designed to only save the eye movement position signals when a visual stimulus is presented. The VE2020 software sends a trigger to the CW6 fNIRS system at the initiation of every visual stimulus presentation. VE2020 sends a digital trigger (50% duty cycle) that is converted to analog via an USB 14-bit digital acquisition (DAQ) card using the range of ±5 Volts (National Instruments USB-6002 Multifunction I/O Device, Austin, TX, USA). The trigger signal is then overlaid with the fNIRS datasets to identify the beginning of the vergence eye movement task and rest periods.

### Experimental design

The experiment utilized a conventional block design of sustained fixation for the ‘off’ stimulus compared to vergence eye movements for the ‘on’ stimulus as shown in Fig. 4B. Prediction is known to decrease the latency and increase peak vergence velocity of eye movements and utilizes additional neural substrates^11,44,52^. Hence, to reduce anticipatory or predictive cues, this experiment utilized a series of vergence eye movements where a random delay between 0.5 and 2 seconds was presented before the initiation of a new vergence eye movement stimulus. The visual stimulus target was an eccentric set of squares (Fig. 4C). This target is important because it gives the participant the perception of depth. Each eye had a set of eccentric squares which when fused would give the participant visual feedback that each set of squares was located at a different depth. Thus, the participant knew via visual feedback when the eccentric squares were fused. Each fixation phase was 16 seconds long, while the eye movements phase was 24 second long. In those 24 seconds, the participant initiated four convergent (inward eye rotation) and four divergent (outward eye rotation) movements. There was a total of 10 rest/task blocks in one session, ending with a rest block. The protocol was repeated for all participants for a second session. The comparison of functional activation within individual participants between the two sessions will assess the reliability for the fNIRS instrumentation using this specific vergence oculomotor task.

### Stereotactic placement of head cap

Unlike an fMRI experiment, an anatomical data set for anatomical markers is not imaged during an fNIRS experiment. To ensure that optodes are reliably placed, the Neural Navigator Stereotactic camera system (Rouge Research Inc.’s Neuronavigations System with BrainSight software, Montreal, Quebec, Canada) is utilized to affix the head cap onto each participant’s scalp. This camera system ensures that functional activity signals are from the FEF, similar to a previous study^26^. Figure 4D–G shows the configured location of the FEF and each optode location. Figure 4D,E show the anatomical locations of sources and detectors. Figure 4F,G show the coronal and axial view, respectively. Both views show the location of the bilateral FEFs used for the study. The Montreal Neurological Institute (MNI) locations for the left (L) and right (R), anterior (A) and superior (S) FEF are (−27L, 17A, 47S) and (27R, 17A, 47S), respectively. This location is within the vicinity of the intersection of the precentral sulcus and superior frontal sulcus reported in other fMRI human studies (Alkan *et al*., 2011; Jaswal *et al*., 2014; Paus^53^; Vernet *et al*.^54^). Figure 4H shows the sensitivity map for the setup derived utilizing the AtlasViewer toolbox in the Homer2 package.

### Data and statistical analysis

The raw fNIRS data were analyzed using two different MATLAB toolboxes: (1) Homer2^55–61^ and (2) NIRS-SPM^62–66^. The Homer2 software was utilized to convert the raw optical intensity into an optical density signal and then hemodynamic concentrations for each source detector channel. These hemodynamic concentrations were processed with a bandpass filter (cutoff frequency range of 0.01 Hz to 0.1 Hz) to remove baseline fluctuations and other physiological measures such as cardiac (1–2 Hz), respiration (0.2–0.4 Hz), and Mayer Waves (~0.1 Hz). The final step in Homer2 preprocessing was the normalizing of data by obtaining the z-scores of the individual channel’s data signal. The start times of each task and rest phases were denoted through the StimGUI of the Homer2 toolbox. These start times were marked based on the analog triggers overlaying the fNIRS data sets that were sent to the CW6 instrument from the VE2020 control program.

An intraclass correlation coefficient (ICC) was calculated for the two sessions for all participants to assess the reliability and reproducibility of fNIRS instrumentation in the FEF region. ICC (1,1) was calculated between each of the 16 channels for each participant^67^. ICC values which were more than one standard deviation away from the mean were considered outliers and hence excluded from the mean group level values. The mean group-level ICC values per channel for all the participants were calculated. A paired t-test was used to compare the ICC values of the HbO and HbR for each participant for each of the four channels studying the bilateral FEFs. Only these two measures were used because they are independent whereas the HbT concentration is a combination of the HbO and the HbR concentration.

The bandpass filtered concentrations obtained through the Homer2 program were utilized for NIRS-SPM version 4. The start times of task and rest phases were also obtained from the StimGUI portion of the Homer2 software. The MNI coordinates of the optode locations obtained from the BrainSight software were utilized to map the channels and its corresponding hemoglobin concentrations to a model cortex included in the SPM8 toolbox. Statistical analysis on the constructed map was accomplished by using a general linear model (GLM). The GLM was computed by modeling the functional activity to the experimental task condition using the NIRS-SPM toolbox defaults settings unless otherwise noted. This first-level analysis is a within participant analysis where activation at various locations is averaged across the scans for an individual participant. A single scan consists of a series of fixation and eye movement phases. The GLM design matrix utilized a low-pass Gaussian filter with a full width half max (FWHM) of 8 seconds. A wavelet transform is additionally applied through the NIRS-SPM toolbox to remove the uncorrelated noise components from the hemodynamic measures to improve the signal to noise ratio. The pixels which have significant activation with Lipschitz-Killing curvature based expected Euler characteristics for a p-value equal to 0.05 correction were plotted on generic anatomic maps provided by the NIRS-SPM toolbox.

Frequency power spectrum analysis was performed by obtaining the discrete Fourier transform of the data signals from each of the channels. This power spectrum was computed utilizing the MATLAB (Version 2018) fft function.

## Supplementary information

Supplemental Material.

## Data Availability

Data will be made available upon request.
