# Supplementary material for: Reliability of Frontal Eye Fields Activation and Very Low-Frequency Oscillations Observed during Vergence Eye Movements: an fNIRS Study
Source: Sci Rep. 2020 Jan 20;10:712. doi: 10.1038/s41598-020-57597-4 (PMC6971237; doi:10.1038/s41598-020-57597-4)
Supplement: Supplementary file 1 — Supplemental Material. [file 41598_2020_57597_MOESM1_ESM.docx]

**Reliability of Frontal Eye Fields Activation and Very Low-Frequency Oscillations Observed during Vergence Eye Movements: an fNIRS Study**

Chang Yaramothu^1^, Xiaobo Li^1^, Cristian Morales^1^, Tara L. Alvarez^1*^

Table 1A: Participants’ Clinical Information: Part 1

| **Subject** | **Gender** | **Age** (years) | **Stereopsis** | | **Near Point of Convergence** | |
| --- | --- | --- | --- | --- | --- | --- |
|  |  |  | Fine | Coarse | Break | Recovery |
|  |  |  | (arc sec) | (arc sec) | (cm) | (cm) |
| 1 | Male | 27 | 30 | 250 | 4.5 | 5 |
| 2 | Male | 20 | 40 | 250 | 6.5 | 7 |
| 3 | Male | 21 | 20 | 250 | 6 | 7 |
| 4 | Female | 20 | 70 | 250 | 5 | 6 |
| 5 | Male | 22 | 30 | 250 | 8 | 9 |
| 6 | Female | 24 | 30 | 250 | 6.5 | 7 |
| 7 | Male | 23 | 20 | 250 | 1 | 2 |
| 8 | Male | 27 | 40 | 250 | 4 | 6 |
| 9 | Male | 26 | 20 | 250 | 5 | 6 |
| 10 | Male | 26 | 20 | 250 | 7.5 | 8.5 |
| 11 | Female | 22 | 20 | 250 | 4.5 | 5.5 |
| 12 | Female | 28 | 25 | 250 | 6.5 | 8.5 |
| 13 | Female | 22 | 20 | 250 | 3.5 | 4 |
| 14 | Female | 25 | 30 | 250 | 5.5 | 7 |
| 15 | Male | 20 | 40 | 250 | 4 | 4.5 |
| 16 | Male | 34 | 50 | 250 | 5 | 7 |
| 17 | Female | 25 | 25 | 250 | 2.5 | 3 |
| 18 | Male | 22 | 40 | 250 | 3.5 | 4 |
| 19 | Male | 25 | 50 | 250 | 5 | 6 |
| 20 | Female | 29 | 20 | 250 | 4 | 5 |
| Average |  | 24.4 | 32 | 250 | 4.9 | 5.9 |
| Std. |  | 3.5 | 13.2 | 0 | 1.6 | 1.8 |

Table 1B: Participants’ Clinical Information: Part 2

| **Subject** | **Vergence Range** | | | | | | **Near Motor Dominance** |
| --- | --- | --- | --- | --- | --- | --- | --- |
|  | **Base Out (∆)** | | | **Base In (∆)** | | |  |
|  | Blur | Break | Recovery | Blur | Break | Recovery |  |
| 1 | n/a | 45 | n/a | n/a | 18 | 14 | Left |
| 2 | 25 | 35 | 30 | 12 | 16 | 14 | Right |
| 3 | 16 | 35 | 30 | 10 | 16 | 14 | Right |
| 4 | n/a | 30 | 25 | n/a | 16 | 6 | Left |
| 5 | 25 | 30 | 20 | 12 | 16 | 14 | Right |
| 6 | n/a | 35 | 25 | n/a | 16 | 14 | Right |
| 7 | 14 | 25 | 20 | n/a | 14 | 12 | Right |
| 8 | 14 | 16 | 14 | n/a | 12 | 10 | Left |
| 9 | 25 | 40 | 35 | n/a | 12 | 10 | Right |
| 10 | n/a | 20 | 18 | 8 | 12 | 10 | Right |
| 11 | 16 | 25 | 18 | 8 | 12 | 10 | Right |
| 12 | 25 | 35 | 25 | n/a | 10 | 6 | Right |
| 13 | n/a | 40 | 35 | n/a | 16 | 14 | Left |
| 14 | 18 | 30 | 25 | 6 | 12 | 10 | Right |
| 15 | 12 | 40 | 35 | n/a | 25 | 20 | Right |
| 16 | 20 | 30 | 25 | 12 | 14 | 12 | Right |
| 17 | 14 | 25 | 20 | 14 | 14 | 12 | Right |
| 18 | n/a | 25 | 20 | n/a | 16 | 14 | Right |
| 19 | 14 | 20 | 18 | n/a | 12 | 8 | Right |
| 20 | 20 | 30 | 25 | 8 | 14 | 12 | Right |
| Average | 18.4 | 30.6 | 24.4 | 10 | 14.7 | 11.8 |  |
| Std. | 4.7 | 7.5 | 6.1 | 2.5 | 3.1 | 3.2 |  |

Table 2: Frequencies at Maximum Power for the Channels over the FEF on an Individual Basis

| Subject | Channel 2 | Channel 5 | Channel 10 | Channel 13 |
| --- | --- | --- | --- | --- |
| 1 | 0.0163 | 0.0163 | 0.0163 | 0.0163 |
| 2 | 0.0159 | 0.0159 | 0.0159 | 0.0159 |
| 3 | 0.0159 | 0.0159 | 0.0159 | 0.0159 |
| 4 | 0.0167 | 0.0167 | 0.0158 | 0.0167 |
| 5 | 0.0156 | 0.0165 | 0.0156 | 0.0139 |
| 6 | 0.0154 | 0.0163 | 0.0163 | 0.0163 |
| 7 | 0.0164 | 0.0164 | 0.0164 | 0.0164 |
| 8 | 0.0159 | 0.0159 | 0.0159 | 0.0159 |
| 9 | 0.0119 | 0.0165 | 0.0165 | 0.0165 |
| 10 | 0.0159 | 0.0159 | 0.0159 | 0.0160 |
| 11 | 0.0164 | 0.0164 | 0.0155 | 0.0164 |
| 12 | 0.0160 | 0.0160 | 0.0160 | 0.0160 |
| 13 | 0.0165 | 0.0165 | 0.0165 | 0.0165 |
| 14 | 0.0154 | 0.0154 | 0.0154 | 0.0164 |
| 15 | 0.0158 | 0.0149 | 0.0158 | 0.0158 |
| 16 | 0.0160 | 0.0160 | 0.0160 | 0.0160 |
| 17 | 0.0162 | 0.0162 | 0.0162 | 0.0162 |
| 18 | 0.0161 | 0.0161 | 0.0161 | 0.0161 |
| 19 | 0.0161 | 0.0161 | 0.0161 | 0.0161 |
| 20 | 0.0159 | 0.0159 | 0.0159 | 0.0159 |
| Avg | 0.0158 | 0.0161 | 0.0160 | 0.0161 |
| STD | 0.0010 | 0.0004 | 0.0003 | 0.0006 |
